# Antioxidants Improve the Proliferation and Efficacy of hUC-MSCs against H_2_O_2_-Induced Senescence

**DOI:** 10.3390/antiox12071334

**Published:** 2023-06-24

**Authors:** Zhaojuan Zheng, Xia Wang, Liming Ouyang, Wenxia Chen, Lixin Zhang, Yulin Cao

**Affiliations:** 1 State Key Laboratory of Bioreactor Engineering, East China University of Science and Technology, Shanghai 200237, China; y30190360@mail.ecust.edu.cn (Z.Z.); y30200496@mail.ecust.edu.cn (X.W.); y30210456@mail.ecust.cn (W.C.); lxzhang@ecust.edu.cn (L.Z.); 2 Beijing Tang Yi Hui Kang Biomedical Technology Co., Ltd., Beijing 100032, China; caoyl@tangyigroup.com

**Keywords:** human umbilical cord mesenchymal stem cells (hUC-MSCs), nicotinamide mononucleotide (NMN), coenzyme Q10, senescence, cell proliferation, RNAseq

## Abstract

Human umbilical cord mesenchymal stem cells (hUC-MSCs) are broadly applied in clinical treatment due to convenient accessibility, low immunogenicity, and the absence of any ethical issues involved. However, the microenvironment of inflammatory tissues may cause oxidative stress and induce senescence in transplanted hUC-MSCs, which will further reduce the proliferation, migration ability, and the final therapeutic effects of hUC-MSCs. Beta-nicotinamide mononucleotide (NMN) and coenzyme Q10 (CoQ10) are famous antioxidants and longevity medicines that could reduce intracellular reactive oxygen species levels by different mechanisms. In this study, hUC-MSCs were treated in vitro with NMN and CoQ10 to determine if they could reduce oxidative stress caused by hydrogen peroxide (H_2_O_2_) and recover cell functions. The effects of NMN and CoQ10 on the cell proliferation, the mRNA levels of the inflammatory cytokine *TNFα* and the anti-inflammatory cytokine *IL10*, and the differentiation and cell migration ability of hUC-MSCs before and after H_2_O_2_ treatment were investigated. The findings revealed that NMN and CoQ10 reduced H_2_O_2_-induced senescence and increased hUC-MSCs’ proliferation in the late phase as passage 12 and later. The *TNFα* mRNA level of hUC-MSCs induced by H_2_O_2_ was significantly decreased after antioxidant treatment. NMN and CoQ10 all reduced the adipogenic differentiation ability of hUC-MSCs. CoQ10 improved the chondrogenic differentiation ability of hUC-MSCs. Furthermore, NMN was found to significantly enhance the migration ability of hUC-MSCs. Transcriptomic analysis revealed that NMN and CoQ10 both increased DNA repair ability and cyclin expression and downregulated TNF and IL-17 inflammatory signaling pathways, thereby contributing to the proliferative promotion of senecent stem cells and resistance to oxidative stress. These findings suggest that antioxidants can improve the survival and efficacy of hUC-MSCs in stem cell therapy for inflammation-related diseases.

## 1. Introduction

Mesenchymal stem cells (MSCs) are promising cell therapy sources due to their multilinear differentiation, their ability to home to injury sites, and the secretion of cytokines that promote tissue healing and/or modulate immune responses [1,2,3]. They are currently being used clinically for the treatment of degenerative, traumatic, metabolic, and inflammatory diseases. In particular, this is an interesting candidate for the treatment of inflammatory diseases because of its potential to influence cellular activity related to innate and adaptive immune systems through paracrine effects [4,5]. For example, several studies have shown that bone marrow–derived MSCs (BM-MSCs) reduced inflammation and improved graft survival [6]. In addition, by inhibiting oxidative stress and inflammatory response levels, BM-MSCs have also been shown to effectively prevent and treat hepatic ischemia reperfusion injury (IRI) [7].

The efficacy of MSCs for treating inflammation diseases will be affected by two aspects in clinical applications: the original replicative senescence of cells [8,9] and their stress responses to microenvironment shock when cells collected from culture are transplanted into the body [10]. Replicative senescence is the “Hayflick limit,” which refers to the phenomenon in which telomerase activity decreases, telomere length shortens, and the proliferation and replication cycles arrest cells during the continuous passage of cells [11]. Due to the limitation of the self-replication ability of MSCs, the cells will senesce and fail to efficiently migrate to the damaged tissue. For example, studies have shown that adipose-derived mesenchymal stem cells (AD-MSCs) of passages 3–5 became trapped in the lungs after injection and did not migrate to the injury site, and many of the cells disappeared just 2 h after infusion [12]. As for the latter aspect, microenvironment shock is often associated with increased oxygen free radical content in injured or inflammatory tissues, which assaults cells and causes damage or even apoptosis [13,14].

Numerous studies provide evidence for the close relationship between oxidative stress, inflammation, and aging [15,16]. Therefore, the oxidation-inflammatory theory of aging, or oxi-inflamm-aging, has been proposed, according to which aging is a loss of homeostasis due to chronic oxidative stress that primarily affects the regulatory systems, such as the nervous, endocrine, and immune systems. The consequent activation of the immune system induces an inflammatory state that creates a vicious circle in which chronic oxidative stress and inflammation feed on each other, thereby increasing age-related morbidity and mortality [17]. Thus, inflammation and oxidative stress are inextricably interrelated. As a result, lowering the oxygen radical group in the environment is clearly beneficial to improving the therapeutic effect of MSCs [18,19]. Given the important role of oxidative stress in the pathogenesis of aging and many clinical conditions such as diabetes, Alzheimer’s disease, and Parkinson’s disease [20], antioxidants may positively impact the treatment of inflammation and aging-associated diseases. However, further research is needed to assess the true efficacy of therapeutic interventions.

Nicotinamide adenine dinucleotide (NAD) is a multifunctional metabolite existing in all living cells in the form of NAD^+^ or NADH. However, NAD levels in the body decrease with age [21]. Therefore, cellular NAD could be used as a therapeutic target for a variety of metabolic and age-related disorders, as well as for extending longevity [22]. NMN has been widely employed in anti-aging health care as a natural and most direct NAD^+^ supplement, and it was also the first antioxidant studied on MSCs and in animal tests. Recent research has discovered that NMN can efficiently enhance BM-MSC proliferation in vitro and in vivo by up-regulating the expression of the “longevity protein” Sirtuin 1 (SIRT1). As a result, the osteogenic potential of in vitro grown BM-MSCs was increased, but adipogenesis was diminished [23].

Coenzyme Q10 (CoQ10), also known as ubiquitin ketone, is frequently used to treat various ailments, including cardiovascular, metabolic, neurological, and reproductive issues, as well as cancer [24]. According to studies demonstrating its anti-aging, anti-oxidative damage, and anti-diminished cell function properties, CoQ10 significantly reduced the number of senescence-associated-galactosidase (SA-β-Gal)-positive cells and prevented the expression of genes associated with aging-related secretory phenotypes. It can also prevent apoptosis, mitochondrial function reduction, and endothelial cell senescence by decreasing the formation of intracellular reactive oxygen species (ROS) [25]. It has also been shown that pre-incubating human umbilical vein endothelial cells (HUVEC) with CoQ10 prevents H_2_O_2_-induced premature senescence and ameliorates the decline in endothelial cell physiological function [25].

In addition to being used in cellular antioxidant studies, these two compounds are also frequently prescribed as anti-aging and longevity medications [26,27]. Both show anti-inflammatory properties and reduce oxidative stress levels in different types of cells, but their effects on anti-oxidation, anti-inflammation, and proliferation in human umbilical cord mesenchymal stem cells (hUC-MSCs) have not been thoroughly investigated. According to the latest research results published by ClinicalTrials.gov, hUC-MSCs have become the second most common MSCs used in clinical trials after BM-MSCs due to their easy acquisition and low-immunogenicity advantages [28].

Therefore, this study aimed to comprehensively evaluate the effects of NMN and CoQ10 on hUC-MSCs in vitro with respect to aging, proliferation, differentiation, secretion, migration, etc., in order to provide evidence for the clinical application of antioxidants as aids in stem cell therapy.

## 2. Materials and Methods

### 2.1. Cell Isolation and Culture

Fresh umbilical cords were obtained from informed, consenting mothers at the First People’s Hospital of Nanjing (China) and rapidly processed. The umbilical cords were cut into small segments and unfolded along the umbilical vein and artery. The colloids on the surface of the umbilical vein and umbilical artery were torn, the blood vessels were removed, and the Wharton jelly was obtained. We cut the colloid into small pieces for the isolation and cultivation of umbilical cord mesenchymal stem cells [29]. The cells were grown in a mesenchymal stem cell serum-free medium (Tofflon Biological Reagent Co., Ltd., Shanghai, China) at 37 °C, 5% CO_2_. The medium was changed every two or three days. When a large number of isolated spindle cells appeared at the edge of tissue blocks, the tissue fragments were removed and the remaining cells continued to be cultured. When the cell confluency exceeded 80%, cells were digested with trypsin (Biological Industries, Jiangsu, China) and sub-cultured in serum-free medium in T-flasks (75 cm^2^) at an inoculation cell density of about 3000 cells/cm^2^.

### 2.2. Immunophenotypical Analysis of hUC-MSCs

The primary antibodies used were as follows: fluorescein isothiocyanate (FITC)-conjugated CD73, CD34, phycoerythin (PE)-conjugated CD90, HLA-DR and allophycocyanin (APC)-conjugated CD105, CD45 (ThermoFisher Scientific, Shanghai, China). hUC-MSCs at a density of 1 × 10^6^ cells were phenotyped using flow cytometry. Primary antibodies were briefly mixed in an appropriate volume of flow cytometry staining solution and added to cells. The cells were incubated on ice for more than 30 min in the dark, then washed three times with flow cytometry staining solution and resuspended. The expression and purity of cell surface markers were detected by flow cytometry.

### 2.3. Antioxidants Treatment

NMN and CoQ10 (>99%) were purchased from Shanghai Yuanye Biotechnology Co., Ltd. (Shanghai, China). NMN was dissolved in Dulbecco’s Phosphate Buffered Saline (DPBS), and CoQ10 was dissolved in absolute ethanol and β-cyclodextrin in application. The cells were divided into 5 experimental groups: (1) control group (labeled as NC): untreated hUC-MSCs; (2) H_2_O_2_ group: hUC-MSCs cultured in a medium containing 200 μM H_2_O_2_ for 2 h; (3) antioxidant group: hUC-MSCs cultured in a medium containing NMN and CoQ10 for 24 h; (4) antioxidant post-processing group: hUC-MSCs cultured in a medium containing 200 μM H_2_O_2_ for 2 h, and then cultured in a medium containing NMN and CoQ10 for another 24 h; (5) antioxidant pre-processing group: hUC-MSCs cultured in a medium containing NMN and CoQ10 for 24 h, and then cultured in a medium containing 200 μM H_2_O_2_ for another 2 h. Cells of passages 6, 9, and 12 were used in experiments. Concentration of NMN was set as 0.1, 1, 10 μM, and that of CoQ10 was set as 0.5, 5, 50 μM.

### 2.4. Cell Growth Curve Assay

Viable cell numbers were evaluated by CCK-8 kit (US EVERBRIGHT, Suzhou, China). In brief, hUC-MSCs were seeded at a density of 2.5 × 10^3^/well in a 96-well plate. The cells were then treated with different compounds. Following incubation, 10 μL of CCK-8 solution was added to each well of the plate and incubated at 37 °C for another 2 h. Absorbance was then measured at a wavelength of 450 nm, using a microplate reader (Bio-Rad, USA). All experiments were performed in triplicate.

### 2.5. Cell ROS Level Measurement

The intracellular ROS level was measured using a ROS Assay Kit (Biyuntian Biotechnology, Nanjing, China). The cells were plated into 6-well plates at a density of 1 × 10^5^/well. Cells in each experimental group were induced with 200 μM H_2_O_2_ for 2 h and then incubated in a medium with or without NMN and CoQ10 for 24 h. Cells were washed with Phosphate Buffered Saline (PBS) and stained according to the kit manufacturer’s instructions.

### 2.6. Senescence-Associated β-Galactosidase (SA-β-Gal) Assay

The cellular senescence assay was performed with hUC-MSCs cultures using the Cellular Senescence Assay Kit (Biyuntian Biotechnology, Nanjing, China) according to the manufacturer’s instructions. Firstly, cells were washed three times with PBS and fixed with the fixative provided in the kit for 20 min. Then, the fixed cells were stained with 200 µL of freshly prepared 1 × SA-β-gal detection solution and sealed overnight at 37 °C. Cells were observed for the development of a blue color under a microscope.

### 2.7. qRT-PCR Analysis

After treatment, total RNA was extracted using an RNA Extraction Kit (Accurate Biotechnology (Human) Co., Ltd., Hangzhou, China), following the manufacturer’s instructions. Total RNA was reverse transcribed using the Evo M-MLV Reverse Transcription Master Mix Kit (Accurate Biotechnology). Real-time quantitative RT-PCR analysis was performed using SYBR Green (Accurate Biotechnology). The primer sequences are listed in Table 1.

### 2.8. Differentiation Ability of the hUC-MSCs

To analyze whether the tri-lineage differentiation ability of hUC-MSCs cultured in vitro was affected by the NMN and CoQ10, hUC-MSCs of passage 12 were cultured at 3.3 × 10^4^/cm^2^ in a 12-well plate. After 4 h, NMN and CoQ10 were added. When the cell confluence reached 80–90%, it was replaced with a differentiation induction medium (Saiye Biotechnology Co., Ltd., Guangzhou, China) for 2 weeks. The induction medium was changed every 3 days. After differentiation, cells were fixed with 4% paraformaldehyde for 20 min and stained with Alizarin Red, Red O, and Alcian Blue separately to detect osteogenesis, adipogenic, and chondrogenic differentiation. After three washes in PBS, images were taken under the microscope.

### 2.9. Migration Assay

A scratch assay was performed to evaluate the hUC-MSCs’ migration activity. Treated cells in 12-well plates were scratched with 200 μL pipettes. At 0, 24, and 36 h after scratching, images were taken under an inverted fluorescence microscope to assess the ability of the cells to migrate into the wound area. Cell migration rates were calculated by analyzing images taken at 0, 24, and 36 h after scratching using an image processing program (NIS-Elements). The following is the calculation formula:

$$RS=\frac{S_{t36}-S_{t0}}{S_{n36}-S_{n0}}$$

*RS*: Relative area ratio
*S_t_*_36_/*S_t_*_0_: growth area of cells in the antioxidant treatment group at 36 h or 0 h
*S_n_*_36/_*S_n_*_0_: growth area of cells in the control group at 36 h or 0 h

### 2.10. RNA Sequencing

hUC-MSCs of passage 12 were seeded into T175 flasks and treated with NMN (10 μM) and CoQ10 (50 μM) for 24 h. Serum-free medium was used throughout the process in all groups. The total RNA of the cells was extracted using the Trizol reagent method (Invitrogen, USA). The total amounts and integrity of RNA were assessed using the RNA Nano 6000 Assay Kit of the Bioanalyzer 2100 system (Agilent Technologies, Shanghai, CA, USA). Libraries of amplified RNA for each sample were prepared in accordance with the Illumina protocol. After the library was qualified, the different libraries were pooled according to the effective concentration and the target amount of data, and then sequenced by the Illumina NovaSeq 6000. The pairing end readings of 150 bp fragments were generated (Novogene, Beijing, China).

### 2.11. RNAseq Data Analysis

Differential expression analysis of two conditions/groups (two biological replicates per condition) was performed using the DESeq2 R package (1.20.0). padj ≤ 0.05 and |log2(foldchange)| ≥ 1 were set as the threshold for a significantly differential expression. Gene Ontology (GO) and KEGG pathway enrichment analysis of differentially expressed genes was implemented by the ClusterProfile R package (3.8.1), in which gene length bias was corrected. GO terms and KEGG pathways with a corrected P value less than 0.05 were considered significantly enriched by differential expressed genes. Protein-Protein interaction network (PPI) analysis of differentially expressed genes was based on the STRING database (https://www.string-db.org/ accessed on 1 June 2023), which is available to known and predicted Protein-Protein Interactions. An interaction with a combined score of ≥0.4 was considered statistically significant. The hub genes of the upregulated or downregulated DEGs were identified with the cytoHubba app (v0.1) from Cytoscape using the degree method.

### 2.12. Statistical Analysis

Data are presented as mean ± standard deviation. Statistical analysis was performed using GraphPad Prism version 9.0.0 software. One-way analysis of variance (ANOVA) or Student’s *t*-test was used to identify differences between groups. A *p* value of <0.05 was considered statistically significant. Image J software was used to analyze the experimental images.

## 3. Results

### 3.1. Characterization of Primary Culture of hUC-MSCs

**Figure 1:**
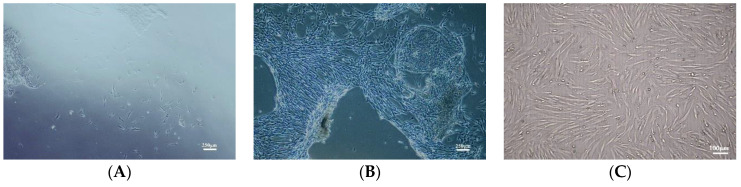
Morphologies of hUC-MSCs. (**A**,**B**) Days 8 and 14 of primary culture for hUC-MSCs Morphology. Scale bar, 250 μm. (**C**) Representative fields of hUC-MSCs morphologies in passage 3. Scale bar, 100 μm.

**Figure 2:**
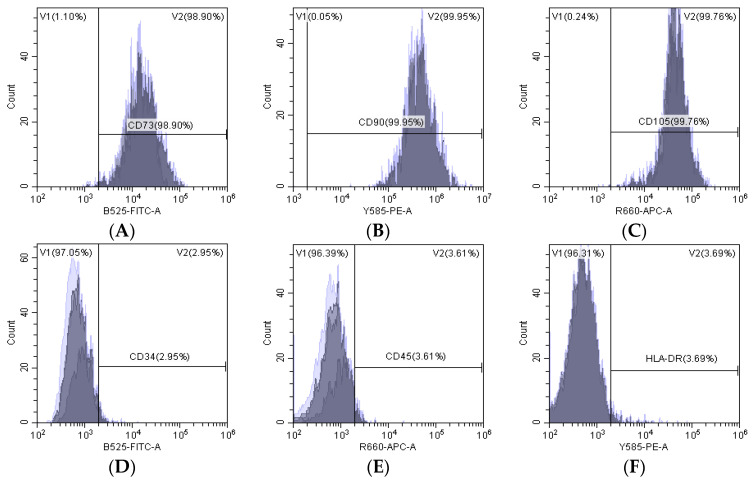
The phenotypic characterizations of hUC-MSCs were identified with flow cytometry. (**A**–**C**) The positive surface markers of hUC-MSCs were CD73 (98.9%), CD90 (99.95%), and CD105 (99.76%). (**D**–**F**) The negative surface markers of hUC-MSCs were CD34 (2.95%), CD45 (3.61%), and HLA-DR (3.69%).

**Figure 3:**
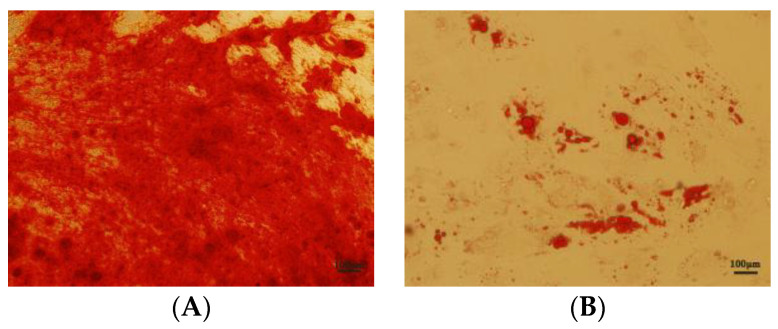
Osteogenic and adipogenic differentiation of MSCs. (**A**) Alizarin red staining. (**B**) Oil red O staining. Scale bar, 100 μm.

The tissue from the cut umbilical cord was cultured in serum-free medium XFM. Some cells migrated out of the tissue block on day 8 (Figure 1A), and on day 14, a large number of cells migrated out of the tissue block (Figure 1B). The tissue block was then removed, and cells were cultured continuously. hUC-MSCs from early passages were found to show a fibroblast-like shape (Figure 1C). The results of the cytometry analysis showed that CD73, CD90, and CD105 were expressed in 98.90%, 99.95%, and 99.76%, respectively, and CD34, CD45, and HLA-DR were expressed in 2.95%, 3.61%, and 3.69%, respectively, (Figure 2A–F) in hUC-MSCs of passage 6. The hUC-MSCs were found to have a multipotent ability to differentiate into osteoblasts and adipocytes cells upon induction (Figure 3A,B). This was consistent with the MSCs’ identification criteria proposed by the International Association for Cell Therapy [28].

### 3.2. ROS Levels Increased in Late Passages of hUC-MSCs Cultured in a Serum-Free Medium

**Figure 4:**
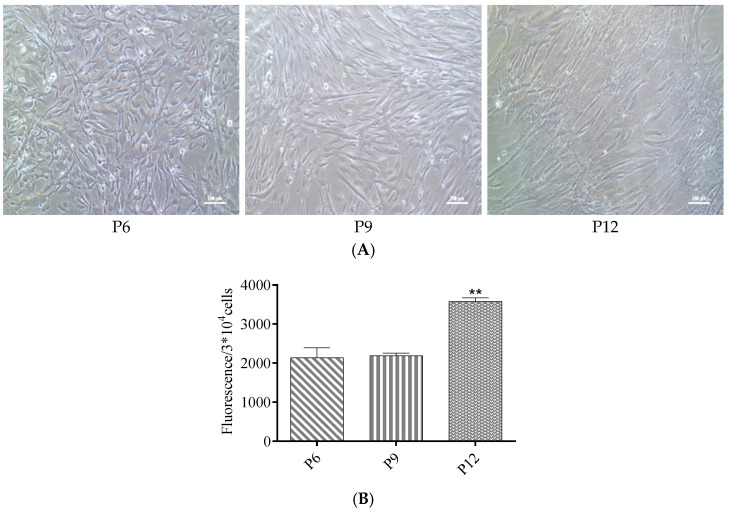
ROS levels and cell morphology of cells in serum-free medium. (**A**) From left to right, microscopic morphology of passages 6, 9, and 12. (**B**) The levels of ROS in hUC-MSCs of passages 6, 9, and 12. Data are presented as mean ± SEM. ** *p* < 0.01 compared with the passages 6 and 9 group. Scale bar, 200 μm.

The cells were continuously cultured in a serum-free medium from primary isolation to passage 12. Cells of early passages displayed a small, spindle-shaped morphology. Cells after passage 11 showed a flatter, larger, and more irregular cell morphology (Figure 4A). Intracellular ROS levels in passages 6, 9, and 12 were detected using a luciferase spectrometer. The results showed that ROS levels increased with the increase of cell passages in late passages, and the ROS level increased 67% in cells of passage 12 compared with those of passages 6 and 9 (Figure 4B).

### 3.3. The Proliferative Capacity Increased in Late Passages of hUC-MSCs after Treatment with NMN and CoQ10

**Figure 5:**
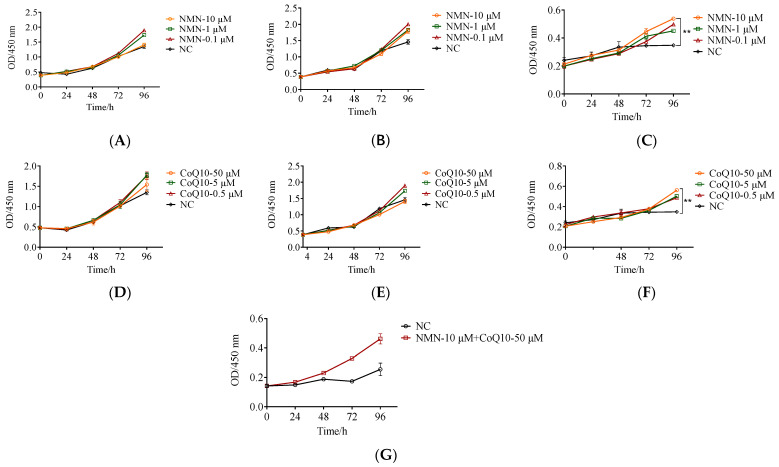
(**A**–**C**): Effects of different concentrations of NMN on cell growth at passages 6, 9, and 12. (**D**–**F**) Effects of different concentrations of CoQ10 on cell viability at passages 6, 9, and 12. (**G**) Effect of combined use of NMN and CoQ10 on cell growth at passages 12. Data are presented as mean ± SEM. Detect the absorbance value at OD450 nm, and the OD value is positively correlated with the number of surviving cells. ** *p* < 0.01 compared with the control group.

To investigate how NMN and CoQ10 acted on the proliferation ability of hUC-MSCs, the medium containing NMN (0.1, 1, 10 μM) and CoQ10 (0.5, 5, 50 μM) was used to culture cells. Cell proliferation ability was determined using the CCK-8 kit. In Figure 5, the ordinates show the OD value from the CCK8 test, which represents the viable cell numbers during culture time. From the OD value and the slope representing the rate of proliferation, the decrease in the proliferation of P12 compared to P6 and P9 can be seen clearly (Figure 5A–C or Figure 5D–F). NMN and CoQ10 did not significantly promote the proliferation of hUC-MSCs in passages 6 (Figure 5A,D) and 9 (Figure 5B,E), but they all significantly promoted that of the passage 12 cells. Compared to the control, NMN (10 μM), and CoQ10 (50 μM) administration boosted cell proliferation by 36% and 47%, respectively (Figure 5C,F). In addition, we found that the combined use of NMN and CoQ10 improved cell proliferation by 55.2% (Figure 5G). Therefore, subsequent experiments were carried out using cells of passage 12, with NMN of 10 μM and CoQ10 of 50 μM.

### 3.4. Cell Migration Ability Increase after Treatment with NMN

**Figure 6:**
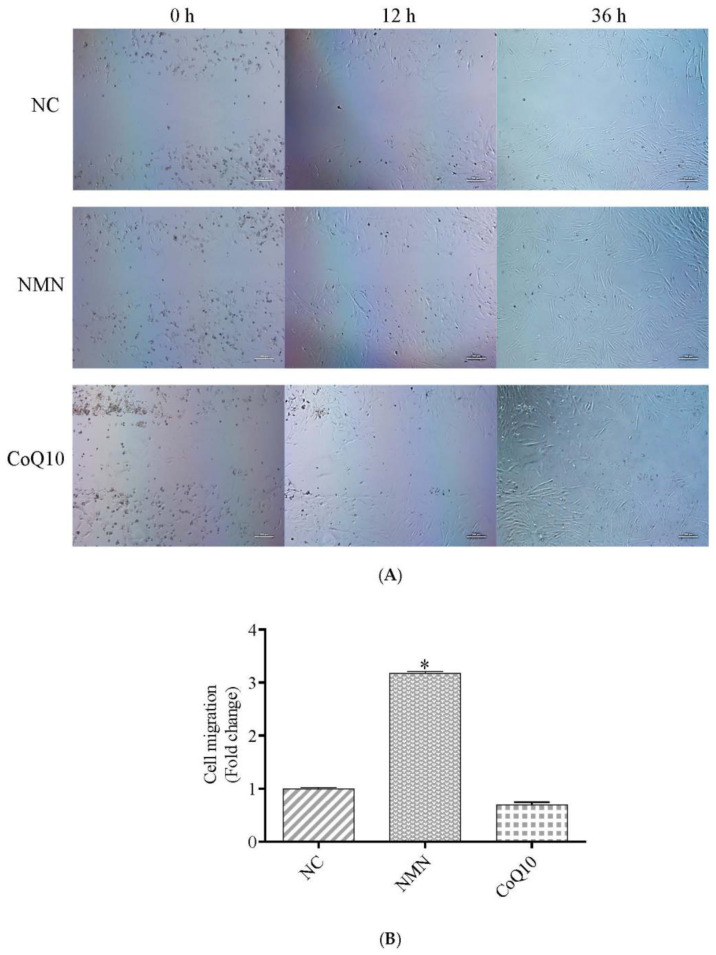
Effects of NMN and CoQ10 treatments on cell migration ability. (**A**) Cell migration images at 4 h, 24 h, and 36 h for control, NMN, and CoQ10-treated cells. (**B**) Fold change in migration activity after 36 h in comparison to control cells. The result is calculated according to the formula presented in 2.9. Data are presented as mean ± SEM. * *p* < 0.05 compared with the control group. Scale bar, 750 μm.

Many studies have shown that mesenchymal stem cells can selectively home to damaged sites in various tissues [30]. However, due to the small number of transplanted MSCs, the efficiency of stem cell homing must be improved. Therefore, we studied the effects of two compounds on the migration ability of stem cells. The results showed that NMN treatment could significantly improve the migration ability of cells (Figure 6A,B).

### 3.5. ROS Levels Decreased after Treatment with NMN and CoQ10

**Figure 7:**
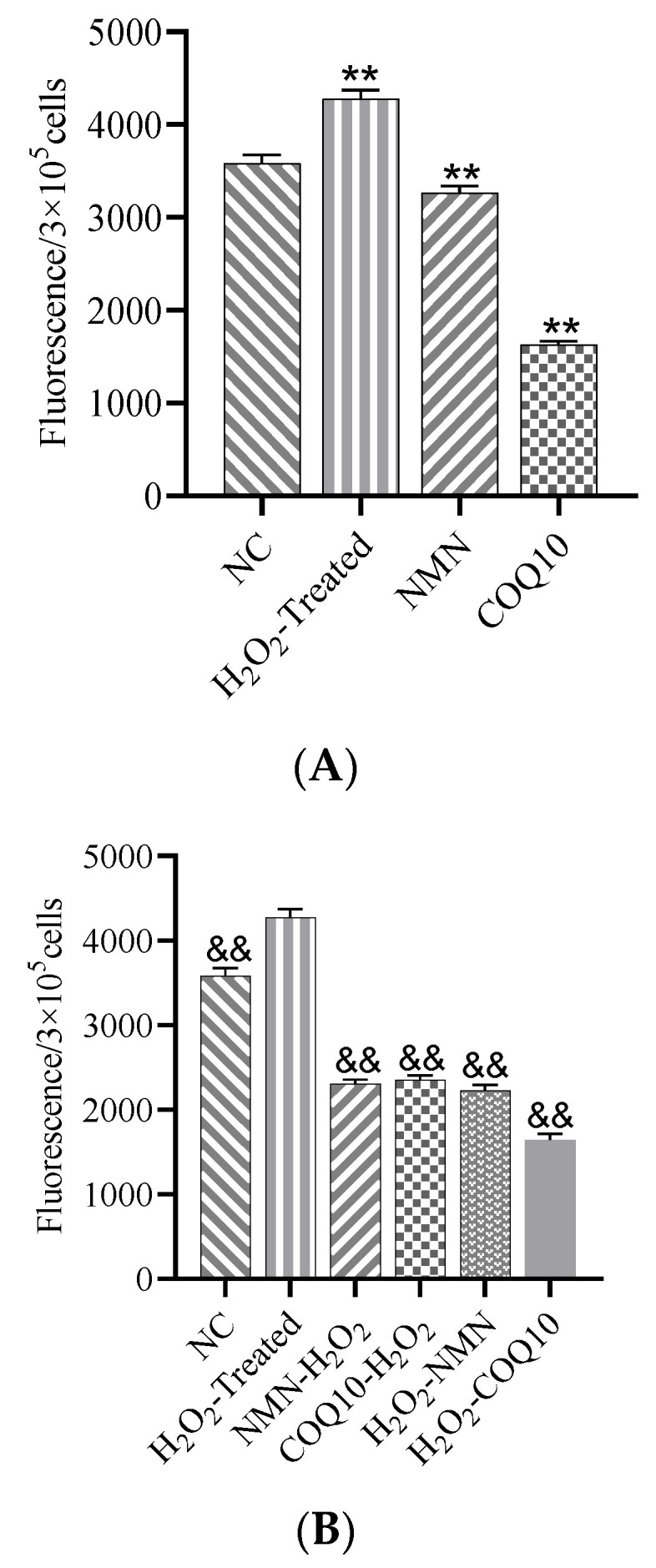
Effects of NMN and CoQ10 treatments on cellular ROS levels. (**A**) NMN and CoQ10 directly acted on cells for 24 h, and cellular ROS levels were determined. (**B**) The ROS level of cells in antioxidant pre-treatment and post-treatment mode. Data are presented as mean ± SEM ** *p* < 0.01 compared with the control (NC) group; ^&&^ *p* < 0.01 compared with the H_2_O_2_-treated group.

Results showed that H_2_O_2_ raised ROS levels significantly, while NMN and CoQ10 significantly reduced the ROS levels of passage 12 hUC-MSCs (Figure 7A). NMN and CoQ10 both reduced the cells’ ROS levels before or after H_2_O_2_ induction for 2 h (Figure 7B).

### 3.6. SA-β-Gal Activity and Senescence-Related Gene Expression Difference after Treatment with NMN and CoQ10

**Figure 8:**
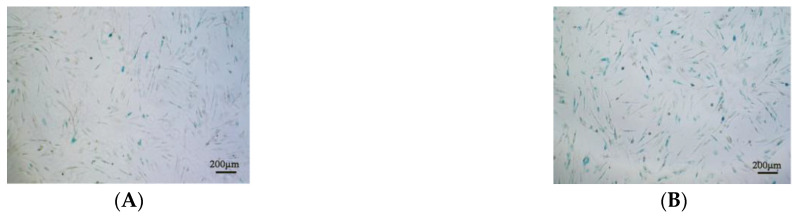

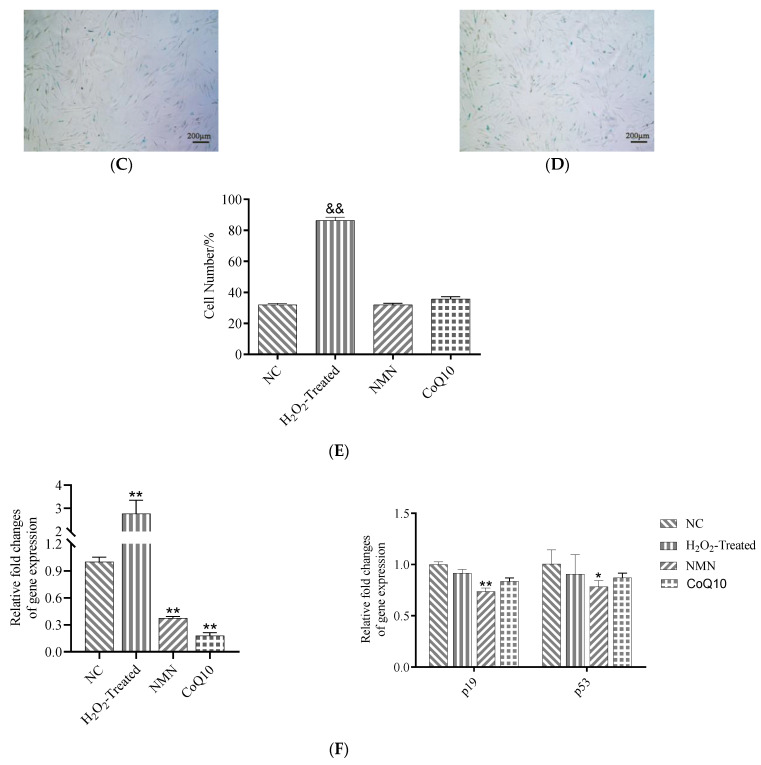
Effects of NMN and CoQ10 treatment on cell senescence. Representative images of SA-β-Gal staining cells of (**A**) the control group, (**B**) the H_2_O_2_-induced treatment group, (**C**) the NMN treatment group, and (**D**) the CoQ10 treatment group. (**E**) SA-β-Gal staining cell ratios of different treatments by image analysis. (**F**) Expression of cellular senescence-related gene *foxe1*, *p19*, and *p53*. Data are presented as mean ± SEM. * *p* < 0.05; ** *p* < 0.01; ^&&^ *p* < 0.01 compared with the control (NC) group. Scale bar, 200 μm.

After H_2_O_2_ treatment for 2 h, about 86% of the cells were positive for SA-β-Gal staining, while there was no significant difference between the staining cell ratios of the antioxidants-treated groups and that of the control (Figure 8A–E). Then, the expression of senescence-related genes was detected, the results showing that the expression of senescence-related gene *foxe1*, *p19*, and *p53* was significantly downregulated after an antioxidant treatment of 24 h (Figure 8F).

### 3.7. Increase of TNFα Gene Expression due to H_2_O_2_ Induction was Significantly Reduced by NMN and CoQ10

As mentioned above, MSCs show two phenotypes of pro-inflammatory and anti-inflammatory cells after transplantation due to the effect of the microenvironment. Similarly, the secreted cytokines also include inflammatory factors such as *TNFα* and anti-inflammatory factors such as *IL10*. Therefore, we also examined the secretion levels of cytokines *IL10* and *TNFα* after antioxidant treatment. The results showed that H_2_O_2_ increased the expression of the inflammation-related factor *TNFα*.

**Figure 9:**
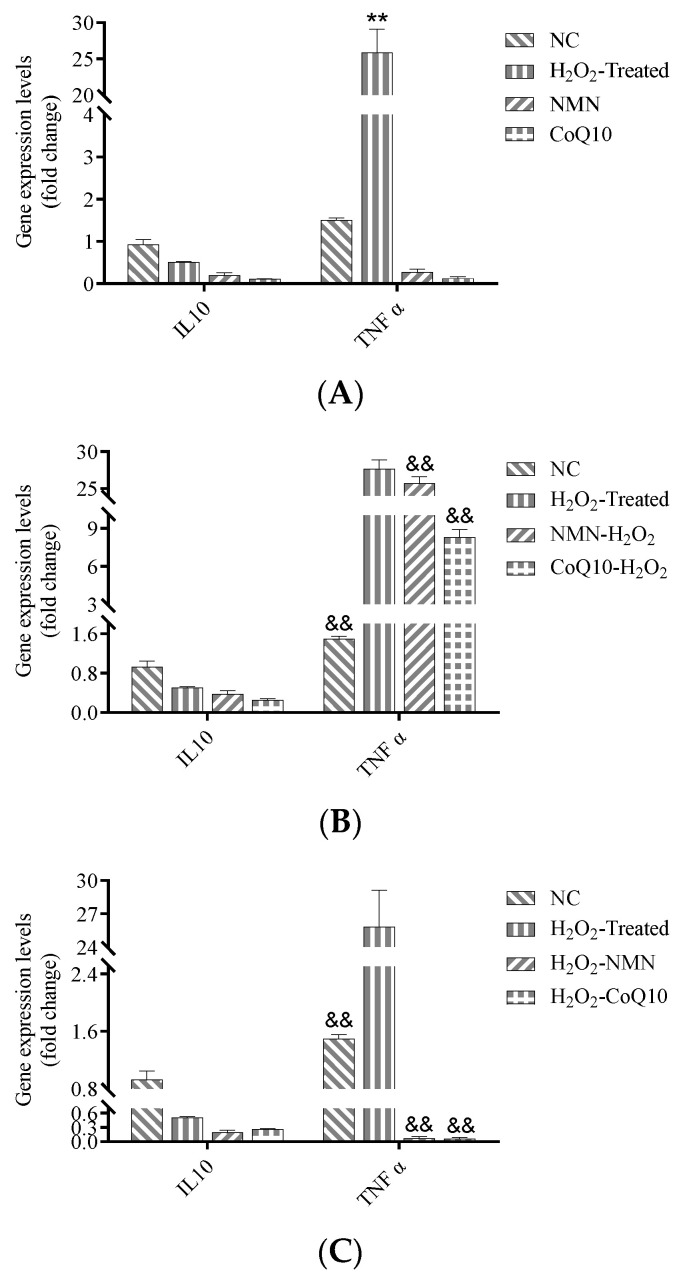
Effects of H_2_O_2_, NMN, and CoQ10 treatments on the expression of inflammation-related cytokine genes. (**A**) Expression levels of *IL10* and *TNFα* after hUC-MSCs were treated with H_2_O_2_ for 2 h or NMN/CoQ10 for 24 h. (**B**) Expression levels of *IL10* and *TNFα* in antioxidant pre-treatment mode. (**C**) Expression levels of *IL10* and TNFα in antioxidant post-treatment mode. Data are presented as mean ± SEM. ** *p* < 0.01 compared with the control group; ^&&^ *p* < 0.01 compared with the H_2_O_2_-treated group.

In contrast, the expression of the anti-inflammatory-related factor *IL10* was not significantly affected by H_2_O_2_ and the antioxidants. Antioxidant treatment alone did not significantly affect these two cytokine genes’ expression (Figure 9A). While in pre-treatment mode, cells were first treated with antioxidants for 24 h followed by H_2_O_2_ induction for 2 h. The expression of the *TNFα* gene was significantly reduced in all three treatment groups compared to the H_2_O_2_ group (Figure 9B). In post-treatment mode, the expression level of *TNFα* was reduced significantly in both treatments compared to the H_2_O_2_ group (Figure 9C).

### 3.8. Effects of NMN and CoQ10 Treatment on Cell Tri-Lineage Differentiation Ability and Expression Genes Related to Stemness and Differentiation

**Figure 10:**
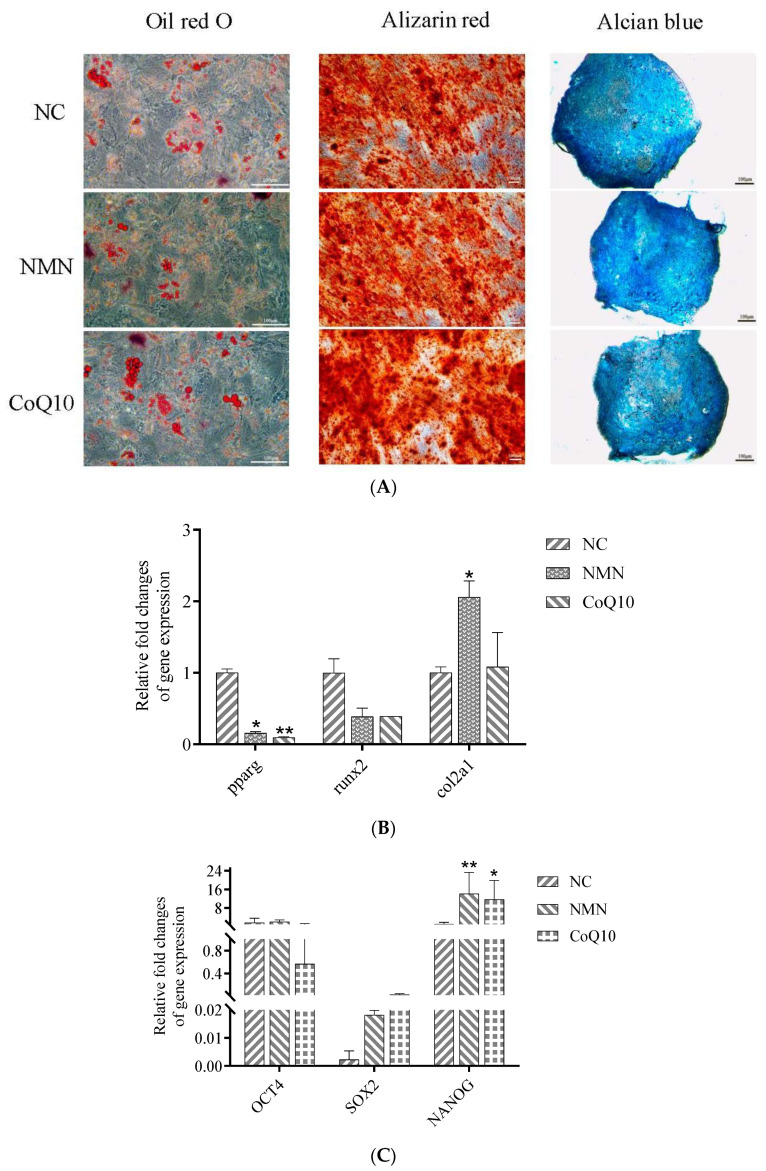
Effects of NMN and CoQ10 treatment on the differentiation potential of cells into three lineages. (**A**) Oil red O, alizarin red, and alcian blue staining of cells in the control group NMN and CoQ10 treatment groups. (**B**) Gene expression levels of adipogenic differentiation-related genes (*pparg*), osteogenic differentiation-related genes (*runx2*), and chondrogenic differentiation-related genes (*col2A1*). (**C**) Gene expression levels of stemness-related genes *oct4*, *sox2*, and *nanog*. Data are presented as mean ± SEM. * *p* < 0.05, ** *p* < 0.01 compared with the control group. Scale bar, 100 μm.

To test the cells’ adipogenic, osteogenic, and chondrogenic differentiation potential, hUC-MSCs were cultured in a complete or differentiation-inducing medium and stained for observation after 2 weeks. The staining results showed that both antioxidants could reduce the adipogenic differentiation ability of cells but not significantly affect the osteogenic and chondrogenic differentiation (Figure 10A). The qRT-PCR results of the two antioxidants on cell adipogenic differentiation and osteogenic differentiation were consistent with the staining results, while NMN treatment increased the expression of the chondrogenic differentiation-related gene *col2A1* (Figure 10B). Treatments with NMN and CoQ10 both enhanced the expression of the stemness-related gene *nanog* (Figure 10C).

### 3.9. Transcriptomic Profiles of hUC-MSCs after NMN/CoQ10 Treatment

**Figure 11:**
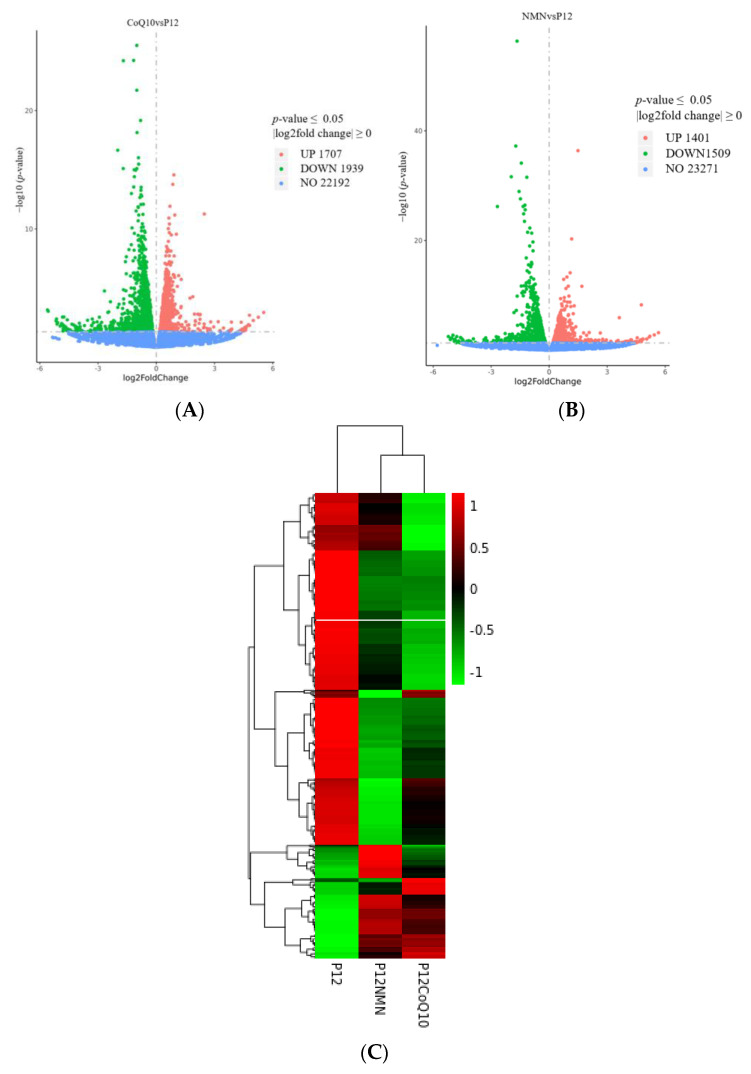
Transcriptomic characteristics of hUC-MSCs in the control group and the antioxidants treatment groups. DEG volcano maps of NMN treatment group versus control (**A**) and CoQ10 treatment group versus control (**B**). Upregulated genes (*p*-value ≤ 0.05, fold change > 1) are shown in red, and downregulated genes are shown in green (*p*-value ≤ 0.05, fold change < 1). (**C**) Heat plot of hierarchical clustering results showing differences between control and antioxidants-treated hUC-MSCs expressing genes (rows). Compared to the control group, the red and green bars indicated genes that were up- and downregulated in the hUC-MSCs in the antioxidants-treated group, respectively.

Transcriptomic analysis of control and antioxidants-treated hUC-MSCs were performed. After treatment with both compounds, the transcriptome underwent a large change compared to the control group. A total of 2910 genes were expressed significantly different in the NMN treatment group, including 1401 upregulated genes and 1509 downregulated genes (Figure 11A). A total of 3646 genes were expressed significantly different in the CoQ10 treatment group, including 1707 upregulated genes and 1939 downregulated genes (Figure 11B). However, the transcriptomes of the two compounds treatment groups were clustered closer than that of the control (hUC-MSCs of passage 12) (Figure 11C). 

### 3.10. Functional Enrichment Analysis of Differentially Expressed Genes in hUC-MSCs in the Antioxidants-Treated Group

A total of 5604 biological process (BP) were enriched in the NMN treatment group, of which 370 were statistically significant with padj < 0.05. Supplementary Figure S1A shows the top 10 significantly enriched BP terms sorted by padj values, which are related to DNA replication, including DNA replication, extracellular matrix organization, sister chromatid isolation, sister chromatid condensation, nuclear mitosis, DNA-dependent DNA replication, etc. The first 10 enriched cellular component (CC) terms include chromosomes, centromere regions, chromosomal regions, extracellular matrix, extracellular matrix components, endoplasmic reticulum lumen, protein extracellular matrix, kinetograne, concentrated chromosomes, etc. Molecular function (MF) terminology is enriched in growth factor binding, ATPase activity, cytokine activity, cell adhesion molecule binding, CXCR chemokine receptor binding, DNA-dependent ATPase activity, ATPase activity coupling, etc. A total of 5,634 BPs were enriched in the CoQ10 treatment group, of which 352 were statistically significant with padj < 0.05. Supplementary Figure S1B shows the top 10 significantly enriched BP terms sorted by padj. As with NMN treatment, enriched BP terms are also associated with DNA replication, and CC ontology is mostly related to chromosomes. Regarding MF ontology, 1,060 MF terms were enriched, and 43 were significant (padj < 0.05). Supplementary Figure S1B shows the first 10 significantly enriched MF terms, and as in the NMN treatment group, the key functions of the altered genes are related to cell adhesion molecule binding, ATPase activity, DNA-dependent ATPase activity, etc.

### 3.11. KEGG Pathway Analysis

**Figure 12:**
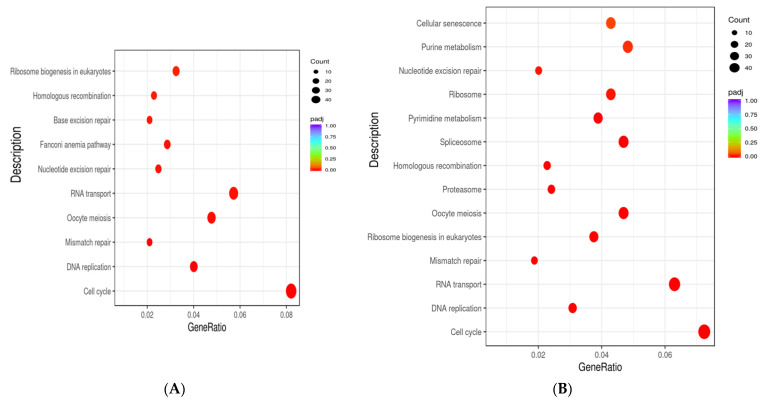
KEGG pathway enrichment analysis for upregulated DEGs in treated groups. (**A**) Significantly enriched (*p* < 0.05) pathways of upregulated DEGs in NMN treatment. (**B**) Significantly enriched (*p* < 0.05) pathways of upregulated DEGs in CoQ10 treatment. GeneRatio represents the ratio of enriched DEGs to total DEGs. The size and color of the dots indicate the number and significance of DEGs enriched in the KEGG pathway, respectively.

The results of KEGG pathway analysis showed that after NMN and CoQ10 treatment, the upregulated genes were enriched in cell cycle, DNA replication, RNA transport and mismatch repair, homologous recombination, nucleotide excision repair, and other DNA repair and replication-related signaling pathways (Figure 12A,B). The marker genes associated with each pathway are shown in Supplementary Table S1.

These results reveal that both NMN and CoQ10 can improve the proliferation capacity of cells by increasing the expression of proteins associated with the cell cycle. Furthermore, by increasing the expression of genes related to DNA repair and replication, they can improve the ability of DNA damage repair, thus improving the ability of cells to resist aging.

**Figure 13:**
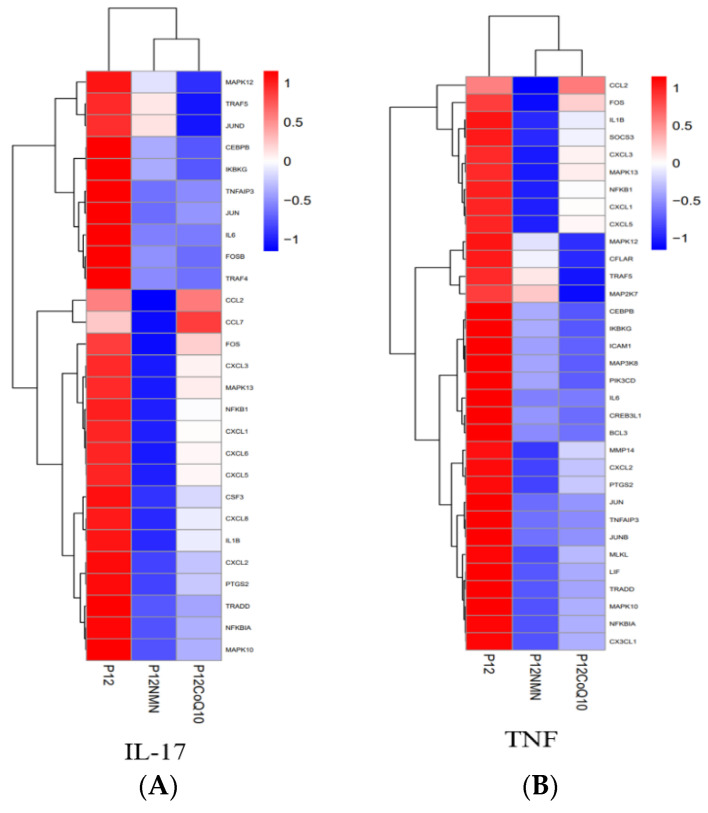
Heat map showing folding changes in genes related to signaling pathways in senescent cells and cells in the antioxidants-treated group. (**A**) Expressive heat map of TNF signaling pathway. (**B**) Expressive heat map of IL-17 signaling pathway. Red indicates an upward adjusted DEG. Blue indicates a downward DEG.

To further explore the antioxidant effects of both compounds, we focused on changes in the spectrum of inflammation-related genes and found that both compounds significantly modulated two inflammation-related pathways: the TNF signaling pathway and the IL17 signaling pathway (Figure 13 and Figure S2).

**Figure 14:**
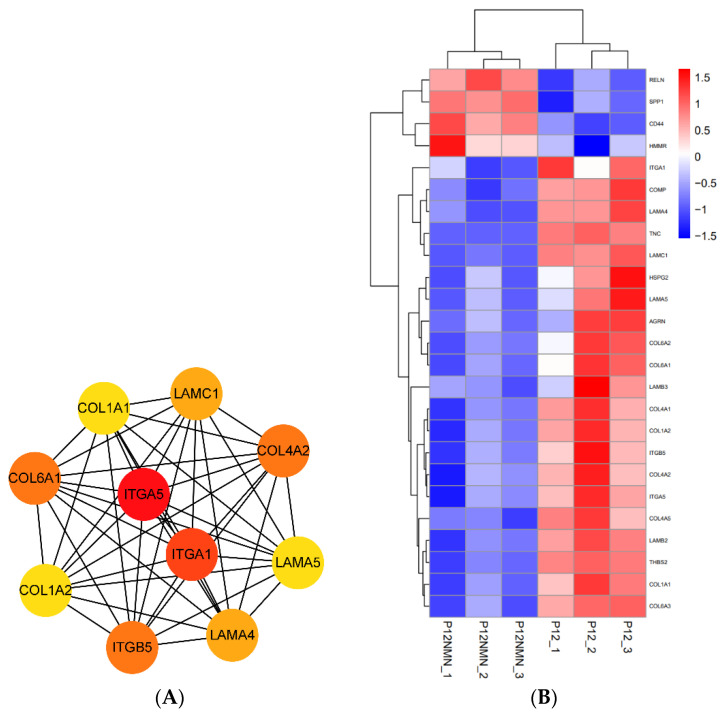
PPI network analysis of ECM receptor signaling pathway in downregulated DEGs in NMN treatment group. (**A**) PPI network analysis based on STRING database. The top 10 hub genes in the network ranked by the cytoHubba degree method were shown. The darker the color, the higher the score and importance of the gene. (**B**) Heat map of DEGs expression enriched in the ECM-receptor interaction pathway. Red colors indicate upregulated DEGs. Blue colors indicate downregulated DEGs.

It was found that NMN also significantly regulated cell surface matrix-related signaling pathways such as ECM-receptor interaction and glycosaminoglycan biosynthesis. NMN significantly downregulated the expression levels of synthygote-related genes (Itga5, Itga1, Itgb5) and significantly downregulated the expression levels of related collagen and laminin-related genes (Col6a, Col4a2, Col1a2, Col1a1, Lama4, Lamc1, Lama5) (Figure 14A,B). Therefore, we speculate that the downregulation of integrinin-related genes and the synthesis of relevant ECM components make the interstitium looser, thereby favoring cell migration.

In addition, there are studies showing that the cell surface distribution of CD44 can restrict the movement of other receptors and affect the organization of the actin cytoskeleton [31,32]. The expression of CD44 on the cell surface favors the migration of cells to the site of the inflammation. Our transcriptome analysis also found that NMN increased CD44 expression in the ECM pathway (Figure 14B). This may be one of the reasons that NMN promotes hUC-MSCs migration.

## 4. Discussion

This study examined the effects of NMN and CoQ10 on hUC-MSCs proliferation, aging, oxidative damage, cytokine secretion, multilineage differentiation, and cell migration. During the H_2_O_2_-induced aging process, the expression of the aging-related gene foxe1 increases. A previous study [33] proved that foxe1 is a novel candidate for senescence. In our preliminary experiments, we found the expression of foxe1 increased steadily with the increase of the passage number of hUC-MSCs. Thus, the expression ratio of foxe1 vs. internal reference gene alas1 was applied as an indication for cell senescence. Studies have shown that tert butyl hydroperoxide can induce the expression of the p16 gene (INK4A)-Rb and p19 (ARF)-p53-p21 (CIP/WAF1) in hematopoietic stem cells [34]. In addition, TERT in aging cells is downregulated, and telomerase activity and telomere length gradually decrease and shorten with cell aging [35]. Both antioxidants increased the proliferation of late passage hUC-MSCs and significantly reduced ROS levels. According to the analysis of RNAseq data, NMN and CoQ10 share similar mechanisms of action on stem cells, as both increase DNA repair capacity and cyclin expression, and downregulate TNF inflammatory signaling pathways, which may be the main reasons for their anti-senescence functions. However, this study also reveals some differences between the two famous antioxidants.

The longevity factor sirtuins consumes NAD to perform multiple functions such as deacetylation, deglutaminase, lipamidase, demethylase, and dessuccinase activity to complete the regulation of longevity, aging, and age-related physiological changes [36]. Therefore, NMN may alleviate cellular senescence by providing a normal supply of NAD for sirtuins to complete its function. Transcriptome results also showed that NMN significantly improved DNA repair capacity and increased the expression level of poly ADP -ribose polymerase (PARP) (Supplementary Table S1), which consumes NAD^+^ to form branched-chain ADP ribose polymers to aid DNA repair. Thus, by enhancing NAD^+^ supply and the expression of PARP, NMN was demonstrated to improve cell proliferation in late generations.

Coenzyme Q10 is an electron carrier in the mitochondrial respiratory chain, a component of the respiratory chain, capable of converting two electrons received from mitochondrial complex I or complex II into alcohol, and then transferring electrons into complex III [37]. Therefore, CoQ10 acts more directly in terms of antioxidation. Studies have shown that CoQ10 could rescue keratinocytes or BM-MSCs by inhibiting ROS production and cleaving pro-apoptotic proteins by lowering inflammation with the essential signaling pathway Nrf2/HO-1 (including caspase-8 and PARP) [38].

In this study, KEGG pathway analysis showed that both compounds significantly downregulated gene expression associated with the activation of the TNF signaling pathway and downstream signaling pathways, including the NF-κB signaling pathway. The downregulation of TNF signaling pathway–related genes by NMN was significantly greater than that of coenzyme Q10 (Figure 13). NMN inhibits the expression of linker proteins TRADD and TRAF2/5, resulting in the weakening of the TNF signaling pathway, as well as lowering the ubiquitination level of NEMO in NEMO/IKK complexes, preventing NF-κB from dissociating from proteasome. Therefore, it directly leads to a decrease in the expression levels of downstream pro-inflammatory factors IL-1b and IL-6, thereby achieving its anti-inflammatory effect (Figure S3). qRT-PCR results showed that TNF-α, the signaling molecule in the upstream of the TNF signaling pathway, decreased in both antioxidants treatment groups (Figure 8), resulting in a weakening of the TNF signaling pathway. The expression pattern of the TNF-α signaling pathway gene from RNAseq was consistent with the qRT-PCR results (Figure 8). These results showed that both NMN and CoQ10 could reduce the level of inflammation in cells and improve the proliferative viability of cells by reducing the expression of genes associated with inflammation.

These findings suggest that when MSCs are exposed to an inflammatory environment, antioxidants may allow them to survive and perform anti-inflammatory functions in vivo. In addition to fighting inflammatory diseases, it may also be beneficial for adjuvant MSCs to improve the treatment of other aging-related diseases.

Studies have shown that the combination of veratrol and coenzyme Q10 can increase the proliferation and viability of human mesenchymal stem cells and promote the differentiation of neural progenitor cells [39]. In this study, we added NMN and CoQ10 to the cell culture process and also found that the combination of NMN and CoQ10 significantly improved cell proliferation viability. Both NMN and CoQ10 can improve mitochondrial function and reduce oxidative stress in cells. Studies have shown that neurodegenerative diseases are caused by mitochondrial dysfunction resulting from increased oxidative stress in cells [40]. Therefore, we speculate that the combination of NMN and CoQ10 may have a similar effect to the combination of resveratrol and CoQ10, which can improve the differentiation ability of nerve cells and alleviate neurodegenerative damage.

In addition, these two antioxidants had different effects on MSCs differentiation and migration. Our results showed that the use of NMN and CoQ10 could decrease the ability of cells to differentiate into adipocytes. It was reported that young hUC-MSCs showed increased osteogenic capacity and decreased adipogenic capacity compared to senescent cells [41]. Thus, the decreased adipogenic ability of hUC-MSCs should be consistent with their rejuvenation. Recent research has indicated that NMN increases osteogenesis and inhibits adipogenesis in aging MSCs via the alteration of the SIRT1 pathway [23]. It also supports SIRT1’s role in balancing MSC quiescence and proliferation, preserving MSCs’ self-renewal capacity, and preventing age-related adult stem cell function decline [42].

This study also initially discovered that NMN promotes MSC migration. RNAseq data suggest that NMN is associated with the ECM signaling pathway in terms of cell migration. We speculate that NMN promotes cell migration, in part due to the downregulation of ECM component expression. The results of this study provide evidence-based support for NMN as a clinical adjuvant to promote the migration of MSCs to the site of damaged tissue after injection. The results indicate that the antioxidants can enhance the stress resistance of MSCs against oxidation and aging and may improve the survival and anti-inflammatory activity of MSCs in vivo.

These two antioxidants are also well-known health supplements and well-liked medications for extending life [43,44]. The study findings discussed here show their ability to slow down stem cell aging and imply that they may contribute to longevity by helping stem cell anti-senescence and anti-inflammatory in vivo.

## 5. Conclusions

NMN and CoQ10 reduced H2O2-induced senescence and increased hUC-MSCs proliferation in the late phase at passage 12 and later. The TNFα expression level of hUC-MSCs induced by H2O2 was significantly decreased after antioxidant treatment. NMN and CoQ10 both reduced the adipogenic differentiation ability of hUC-MSCs. CoQ10 improved the chondrogenic differentiation ability of hUC-MSCs. Furthermore, NMN was found to significantly enhance the migration ability of hUC-MSCs. Transcriptomic analysis revealed that NMN and CoQ10 both increased DNA repair capacity and cyclin expression and downregulated TNF and IL-17 inflammatory signaling pathways, thereby contributing to the proliferative promotion of senescent stem cells and resistance to oxidative stress. These findings suggest that antioxidants can improve the survival and efficacy of hUC-MSCs in stem cell therapy for inflammation-related diseases.

## Figures and Tables

**Table 1:** Primers used for qRT-PCR of target gene in this study.

Gene Name	Primer	Sequence (5′-3′)	Accension Number
*foxe1*	Forward Reverse	GGAGTGTTCAGATGGACTGTTT CAGCCCTTCCCTGTTAAGTTTAT	NM_004473
*p19*	Forward	AGTCCAGTCCATGACGCAG	NM_001800.4
	Reverse	ATCAGGCACGTTGACATCAGC	
*p53*	Forward	CGCTTCGAGATGTTCCGAGA	NC_000017.11
	Reverse	CTGGGACCCAATGAGATGGG	
*pparg*	Forward Reverse	GAAGACGGAGACAGACATGAG GCAACTGGAAGAAGGGAAATG	NM_001354666
*runx2*	Forward Reverse	ATGTCCGCCACCACTCACTACC TGGAGTGCTGCTGGTCTGGAAG	NM_001024630
*col2a1*	Forward Reverse	TGAACCTGGACGAGAGGGAAGC ACAGCACCAGTCTCACCACGAT	X16711
*oct4*	Forward	CTTGCTGCAGAAGTGGGTGGAGGAA	NM_001285986.2
	Reverse	CTGCAGTGTGGGTTTCGGGCA	
*sox2*	Forward	TGGACAGTTACGCGCACAT	NM_003106.4
	Reverse	CGAGTAGGACATGCTGTAGGT	
*nanog*	Forward	AATACCTCAGCCTCCAGCAGAT	NM_024865.4
	Reverse	TGCGTCACACCATTGCTATTCTT	
*IL10*	Forward Reverse	CTGGGAGCCCACACTATTTATT GATGCACCACTACCTTTCTACC	NM_001382624
*TNF* *α*	Forward Reverse	GGAGTGTTCAGATGGACTGTTT CAGCCCTTCCCTGTTAAGTTTAT	NM_000594
*alas1*	Forward Reverse	CCTGGATGGATGAGTGGCTTCT AATGGGCAGCGGCGAACAA	

## Data Availability

The data used to support the findings of this study are available from the corresponding authors upon request. The RNA-seq data have been deposited in NCBI’s Gene Expression Omnibus and are accessible through GEO Series GSE226464 (https://www.ncbi.nlm.nih.gov/geo/query/acc.cgi?acc=GSE226464 accessed on 1 June 2023).

## Supplementary Materials

The following supporting information can be downloaded at: https://www.mdpi.com/article/10.3390/antiox12071334/s1. Figure S1: Functional classification of GO enrichment clusters. Figure S2: KEGG pathway enrichment analysis for down-regulated DEGs in treated groups. (A) The down-regulation of DEGs in the NMN treatment group significantly enriched the first 20 signaling pathways. (B) The down-regulation of DEGs in the CoQ10 treatment group significantly enriched the first 20 signaling pathways. Figure S3: Analysis of changes in gene expression associated with TNF signaling pathway by KEGG enrichment. Red indicates down-regulation of gene expression levels. Table S1: Symbol genes involved in KEGG pathways associated with DNA replication and repair.
