# Supplementary material for: Antioxidants Improve the Proliferation and Efficacy of hUC-MSCs against H2O2-Induced Senescence
Source: Antioxidants (Basel). 2023 Jun 24;12(7):1334. doi: 10.3390/antiox12071334 (PMC10376626; doi:10.3390/antiox12071334)
Supplement: Supplementary file 1 [file antioxidants-12-01334-s001.zip › antioxidants-2375425-supplementary.pdf]

KEGG ID	Term	NMN-Symbols	CoQ10-Symbols
hsa03030	DNA replication	MCM3/MCM6/MCM4/POLA1/RFC3/RPA1/POLD3/MCM2/PCNA/PRIM1/POLE3/DNA2/PRIM2/POLE/FEN1/MCM5/RFC4/MCM7/POLE2/RFC1/RFC2	MCM3/PCNA/MCM6/MCM4/MCM2/RPA1/RFC3/PRIM1/MCM7/POLE3/MCM5/POLD3/FEN1/RPA2/RFC4/POLA1/RFC5/DNA2/PRIM2/RFC2/RPA3/POLE2/POLA2
hsa04110	cell cycle	MCM3/MCM6/MCM4/RBL1/ANAPC1/ORC1/MCM2/PCNA/ESPL1/SMC3/PRKDC/CDK1/CDC7/BUB3/STAG2/E2F2/SKP2/BUB1B/BUB1/DBF4/YWHAH/STAG1/CDC6/SMC1A/RAD21/PKMYT1/CDC25B/MCM5/MAD2L1/YWHAQ/YWHAZ/CDK6/WEE1/GSK3B/MCM7/CDKN2D/ORC3/CCNB2/TFDP1/ANAPC10/SMAD2/CCNE2/CCNB1	MCM3/PCNA/MCM6/MCM4/MCM2/CDC6/MCM7/ORC1/RBL1/MCM5/YWHAQ/CCNB1/CDC7/SMC3/CDK1/CCNB2/BUB1B/YWHAZ/STAG2/CDC25A/YWHAH/ORC3/BUB3/DBF4/MAD2L1/SMC1A/E2F2/TTK/PRKDC/STAG1/TFDP1/CDK2/YWHAE/RBX1/ORC5/E2F1/PTTG1/SKP1/CDC45/CCNA1/BUB1/ESPL1/CHEK1/RAD21/YWHAG/ANAPC10/YWHAB/ORC6/ANAPC1/SMAD2/MAD2L2/PKMYT1/HDAC2/CCNE2
hsa03430	Mismatch repair	MSH2/RFC3/RPA1/EXO1/POLD3/PCNA/MSH6/RFC4/MLH3/RFC1/RFC2	PCNA/RPA1/RFC3/MSH2/EXO1/POLD3/RPA2/RFC4/RFC5/MSH6/MLH1/RFC2/MLH3/RPA3
hsa03420	Nucleotide excision	RFC3/RPA1/POLD3/PCNA/POLE3/POLE/	PCNA/RPA1/RFC3/POLE3/POLD3/RPA2/

	repair	GTF2H1/RFC4/POLE2/ GTF2H3/CUL4B/RFC1/RFC 2	RFC4/RFC5/MNAT1/ GTF2H1/RBX1/RFC2/ RAD23B/RPA3/POLE2
hsa03013	RNA transport	NUP50/NDC1/EIF1AX/ EIF3C/EIF2S2/NUP107/ NUP155/EIF5/NUP205/ KPNB1/EIF2S3/RAN/TGS1/ GEMIN5/SEH1L/XPO1/NU P160/ RPP30/EIF2S1/EIF3E/NUP9 8/ NUP153/SUMO1/NUP43/ NUP133/XPO5/NUP88/ FMR1/NCBP1/STRAP	NDC1/NUP155/NUP50/ RAN/EIF1AX/SUMO3/ NUP133/NUP88/NUP10 7/ SEH1L/EIF2S1/KPNB1/ EIF3E/TGS1/RBM8A/ EIF2B3/DDX20/EIF5/ SUMO1/ELAC2/STRAP / GEMIN5/RPP30/EIF2S3 / EIF4E/EIF2S2/NUP160/ POP1/XPO5/SUMO2/ NUP85/NUP98/EIF3H/ NUP37/NCBP2/GEMIN 4/ NUP54/XPO1/EIF3A/ NUP205/CLNS1A/ RPP38/NXT2/FXR1/ GEMIN2/FMR1/EIF5B
hsa03440	Homologo us recombina tion	BRCA2/BRCA1/RPA1/ BARD1/BLM/POLD3/ RAD54L/XRCC2/RBBP8/ ABRAXAS1/MRE11/BRIP1	RPA1/BLM/POLD3/ RPA2/BRCA1/BRCA2/ RAD54L/RBBP8/BRCC 3/ BARD1/XRCC2/TOPBP 1/ ABRAXAS1/RPA3/ MRE11/NBN/RAD51C
hsa03410	Base excision repair	POLD3/PCNA/POLE3/ POLE/FEN1/NEIL3/UNG/ POLE2/PARP4/HMGB1/PA RP1	PCNA/POLE3/POLD3/ HMGB1/FEN1/UNG/ PARP2/NEIL3/PARP1/ POLE2

Supplementary Table S1 : Symbol genes involved in KEGG pathways associated with DNA replication and repair.

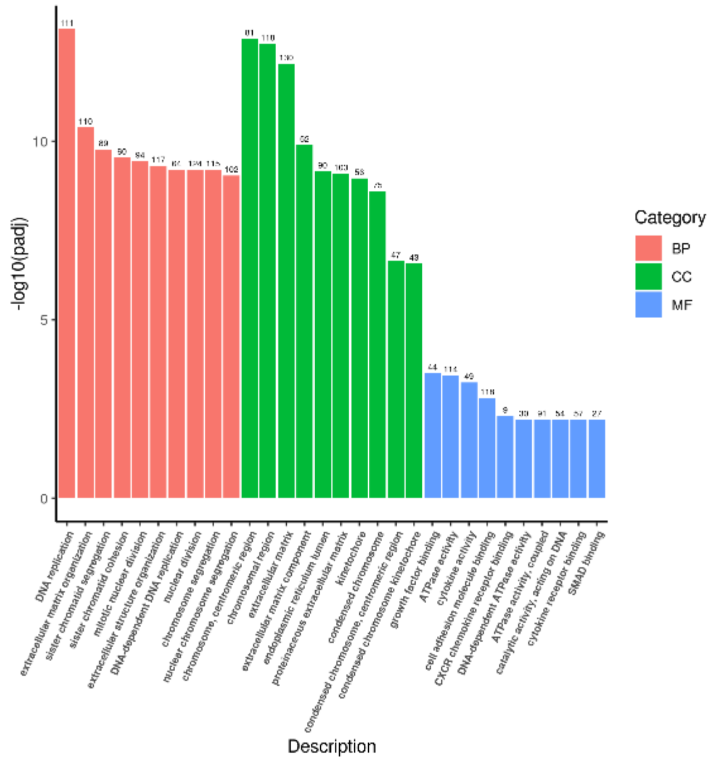

(A)

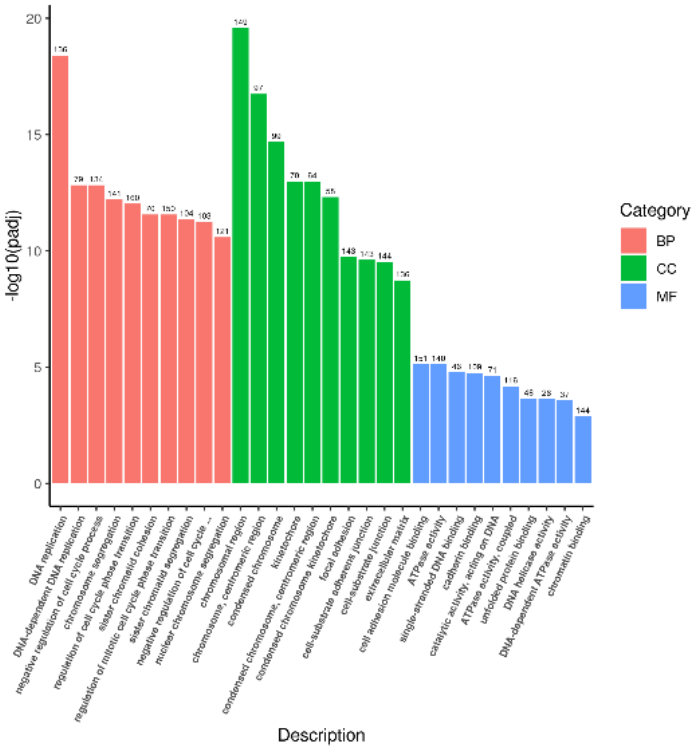

(B)

Supplementary Figure S1: Functional classification of GO enrichment clusters.

According to ontology classification, biological process (BP), cellular component (CC), and molecular function (MF), the distribution of GO terms for DEG between the control and drug treatment groups uc-MSCs is annotated. (A) NMN *vs*. P12 GO enrichment analysis plot; (B) CoQ10 *vs*. P12 GO enrichment analysis plot. The number of DEGs associated with each term is shown.

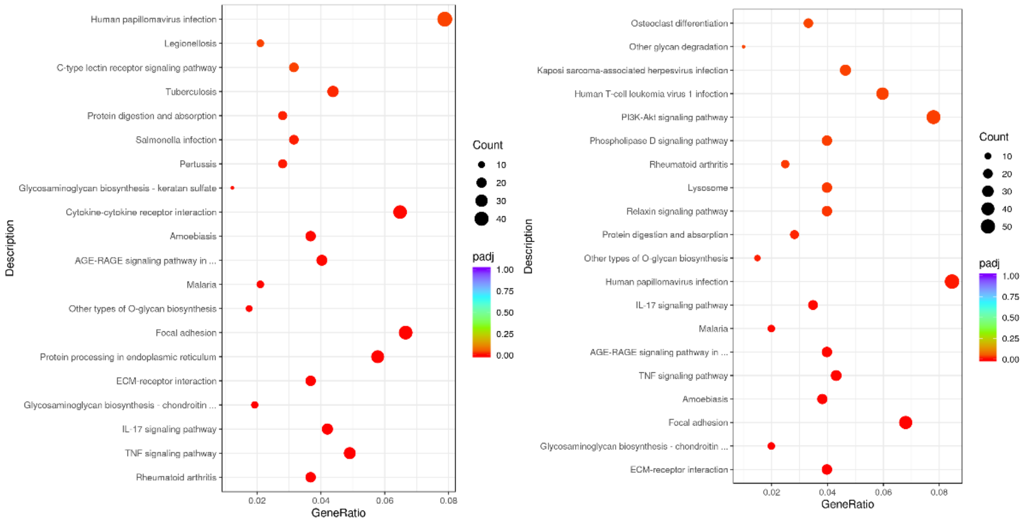

(A) (B)

Supplementary Figure S2: KEGG pathway enrichment analysis for down-regulated DEGs in treated groups. (A) The down-regulation of DEGs in the NMN treatment group significantly enriched the first 20 signaling pathways. (B) The down-regulation of DEGs in the CoQ10 treatment group significantly enriched the first 20 signaling pathways.

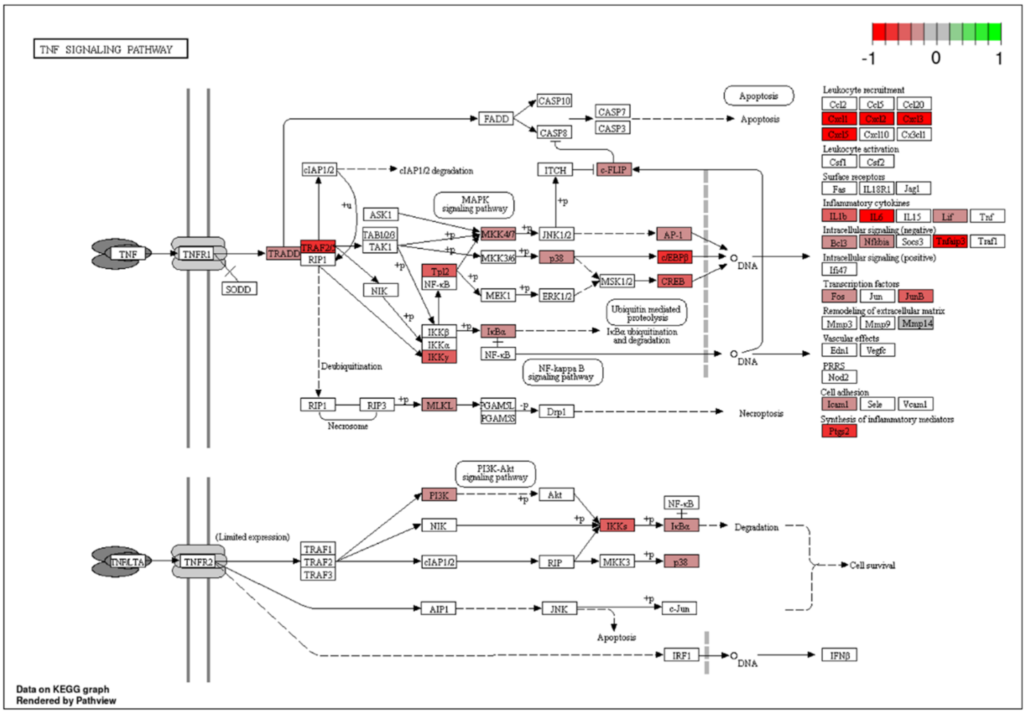

Supplementary Figure S3: Analysis of changes in gene expression associated with TNF signaling pathway by KEGG enrichment. Red indicates down-regulation of gene expression levels.
